# Circulating Autoantibodies to Endothelial Progenitor Cells: Binding Characteristics and Association with Risk Factors for Atherosclerosis

**DOI:** 10.1371/journal.pone.0097836

**Published:** 2014-06-19

**Authors:** Jacob George, Marco Matucci-Cerinic, Iris Bar, Sara Shimoni

**Affiliations:** 1 Heart Center, Kaplan Medical Center, Rehovot, Affiliated to the Hebrew University, Jerusalem, Israel; 2 Department of Experimental and Clinical Medicine, Division of Rheumatology AOUC, University of Florence, Florence, Italy; Medical University Innsbruck, Austria

## Abstract

**Objective:**

Endothelial progenitor cells (EPC) are committed to transform into EC promoting vasculogenic ischemic repair. Anti-endothelial cells (AECA) have been described in various disorders with an associated vascular damage. Herein, we explored a novel circulating population of IgG reactive with EPC, in patients with differential risk profile for atherosclerotic vascular disease.

**Approach and Results:**

A novel cyto-ELISA system was established where the coated cells were late outgrowth EPC. Levels of anti-EPC antibodies were determined in 100 subjects and differential risk score for atherosclerosis, as well as to circulating EPC levels and the inflammatory markers IL-6 and C-reactive protein. To study endothelial cell (EC) activating properties, sera were tested for their ability to induce VCAM-1 expression in a cell ELISA system. Detectable levels of anti-EPC antibodies, that correlated with age, Framingham risk score and CRP concentrations but did not associate with levels of LDL, HDL, hypertension or diabetes, were detected. Anti-EPC antibodies were distinct from EC binding antibodies as shown by competitive inhibition studies, and have been positively correlated with the extent of EC activation manifested by in vitro VCAM-1 expression.

**Conclusion:**

This is the first study showing a newly defined subgroup of self-antibodies binding EPC and associating positively with the Framingham risk score. Further studies are required to characterize and test this interesting subset of EPC binding autoantibodies and their potential significance.

## Introduction

Endothelial progenitor cells (EPC) are a subset of hematopoietic progenitors that circulate in the peripheral blood and play an active role in maintaining the integrity of the endothelium as well as promoting the recovery from various insults that result in tissue ischemia [1], [2]. The number and function of EPC, namely their ability to differentiate into endothelial cells, to produce/secrete a panel of proliferative cytokines and to transmigrate through the endothelial lining determine their vasculogenic capacity.

There are multitude of factors that control the number and function of EPC, thereby dictating their efficacy in promoting tissue healing. These factors include cytokines that promote their proliferation and crossing of the bone marrow barrier as well as those factors facilitating their transmigration to the affected organs and triggering peripheral senescence [1], [2].

The seminal work Asahara et al [3] back in 1997, outlining the presence of circulating EPC, was followed by numerous studies that supported this observation and further demonstrated their presence in various pathological states as well as their therapeutic potential [1]–[4]. Several reports have shown that in patients with risk factors for atherosclerotic vascular disease, the numbers of peripheral EPC are significantly reduced [5], [6]. However, the question of whether this finding is a result or an causal contributor to atherosclerosis has not been resolved.

Autoantibodies to cellular components have been described in several conditions. Examples include anti-heart autoantibodies in patients with post myocardial insults such as pericarditis [7], [8], anti smooth muscle [9] and anti parietal [10] cell antibodies. Of particular interest is the subgroup of autoantibodies reactive with endothelial cells (anti endothelial cell antibodies; AECA) that have been demonstrated in various immune mediated disorders, the most commonly described of which is systemic sclerosis [11], in particular those with pulmonary hypertension [12], where the prevalence of AECA reaches nearly 80%. With regard to atherosclerosis, small studies have not yielded conclusive results as to the prevalence of AECA [12], [13]). This finding has prompted researchers to hypothesize that AECA may not only stand as markers of vascular damage but may also be pathogenic and contribute to the pathological features of the vascular damage [14]. Indeed, there are several reports where AECA from patients with various autoimmune disorders, even those with lower affinity, were found capable of inducing endothelial cell activation *in vitro* [14], [15].

In this study, we explored the possibility that EPC binding antibodies are present in the peripheral circulation. This is the first report documenting the presence of these autoantibodies, their binding properties and their association with risk factors for atherosclerotic vascular disease.

## Methods

We studied 100 volunteers (Table 1), older than 25 years of age (median 61 years (27–90)), with and without conventional atherosclerotic cardiovascular risk factors. Subjects were evaluated for traditional risk factors and the Framingham risk factor score was calculated as described [12]. Subjects with any type of malignant or hematologic disorder or those taking anti-inflammatory agents were excluded. All enrolled subjects underwent a detailed assessment of cardiovascular risk. This study was approved by the Kaplan Medical center institutional ethics committee. All patients provided written informed consent for participating in the study, including the collection and generation of the cell lines.

**Table 1:** Baseline characteristics of the study population.

	N = 100
Age (years)	61 (27–90)
Male/Female	55/45
DM (n)	19
HTN (n)	54
Hyperlipidemia (n)	55
HG (g/dL)	13.56±1.5
WBC	7.02±2
Platelets	279±70
Creatinine (mg/dL)	0.88±0.19
Total cholesterol (mg/dL)	177±34
TG (mg/dL)	128±69
HDL (mg/dL)	51±14
LDL (mg/dL)	100±29
Framingham risk score	8±0.8

DM-Diabetes mellitus, HTN-Hypertension, HG-Hemoglobin, WBC-White blood cells, TG-triglyceride, HDL-high density lipoprotein, LDL-low density lipoprotein.

### ELISA for Detection of AECA and Anti-EPC Autoantibodies

Human umbilical vein endothelial cells (HUVEC) were produced and grown as previously described and serum levels of IgG AECA were determined by ELISA as shown [13].

In brief, HUVEC were seeded in gelatin-coated 96-well microtiter plates at 2.5×10^4^ cells per well and allowed to grow to confluence for 1 or 2 days. Since fixation was required, after washing with fresh growing medium the wells were covered with 100 µl Glutaraldehyde solution (3%, Ph 7.2, Paraformaldehyde 2.5%, Cacodylate buffer 0.1 M) at a final concentration of 0.1% in PBS. Cells were washed 3 times with HBSS and incubated with a blocking buffer (HBSS: 0.5% BSA, 150 µl per well) for 30 min at 37°C to prevent non-specific binding of antibody. After additional washing with HBSS, the endothelial cells were exposed to 100 µl diluted serum (dilutions 1∶25 in HBSS −0.2% BSA) for 90 min at RT (or overnight at 4°C) with slow shaking. Cells were washed again and incubated with 100 µl of the second antibody, alkaline phosphatase-conjugated goat anti-human IgG (Jackson ImmunoResearch Laboratory, WestGrove, PA) diluted 1∶10,000 in HBSS −0.2% BSA for 60 min at RT with shaking. After washing 3 times, 100 µl of 1 mg/ml p-nitrophenyl phosphate in 50 mmol/l carbonate buffer containing 1 mmol/l MgCl2 pH 9.8 was added as a substrate. The reaction was stopped after 30 min by adding 50 µl of 1 mol/l NaOH. The OD was read at 405 nm in an ELISA plate reader (EAR400 AT, SLT-Labinstruments, Austria).

We used late-outgrowth EPC as the target cell population for determination of anti-EPC antibodies as this population most likely reflects true circulating EPC [16]. The blood mononuclear cell fraction from healthy donors was collected by Ficoll (Pancoll, Dutcher, France) density gradient centrifugation and resuspended in endothelial growth medium (EGM-2) (Lonza, Verviers, Belgium). Cells were then seeded on collagen-precoated 12-well plates (BD Biosciences) at 2×10^7^ cells per well and stored at 37°C and 5% CO_2_. After 24 hours of culture, adherent cells were washed once with phosphate-buffered saline and cultured in EGM-2 with daily changes until the quantification. Colonies of endothelial cells appeared between 9 and 26 days of culture and were identified as well-circumscribed monolayers of cells with a cobblestone appearance. The next steps of the ELISA were performed as described for the AECA ELISA.

### Determination of Circulating EPC and Late Outgrowth EPC by FACS

The number of circulating EPCs was assessed by FACS analysis by staining 1 million cells for two-color FACS analysis employing the following monoclonal antibodies: fluorescein isothicyanate-anti-CD34 (IQ products) and allophycocyanin-anti VEGF-receptor 2 (KDR, R&D systems). The various EPC phenotypes assessed were CD34+ and CD34+KDR+.

Late outgrowth EPC were characterized by the expression of several EPC markers: CD34-PE, KDR-APC, CD31-FITC and CD133- biotinylated (Miltenyi biotec), and secondary Rabbit anti mouse PerCP (Santa Cruz).

### Competitive Inhibition of Binding of Anti-EPC Antibodies

In order to determine whether anti-EPC antibodies are actually identical to AECA were performed competitive inhibition studies. For this purpose, we chose several sera with high levels EPC binding antibodies. Sera (1∶25) were incubated with different concentrations of HUVEC overnight and evaluated using the above described anti-EPC ELISA.

### EC Activation In vitro as Determined by VCAM-1 Expression Employing ELISA

HUVEC or Late outgrowth EPC were grown onto 96-well plates washed and fixed with 0.1% glutaraldehyde, and treated with PBS containing 0.2% Triton X-100 to permeabilize the cell membrane. Sera from all subjects diluted 1∶2 were added to the plates overnight. Also, 6 total IgG fractions with HUVEC binding and non HUVEC binding properties were purified from the respective sera using protein G columns and used in concentrations of 10 micrograms/milliliter. Plates were blocked with 3% BSA and incubated with biotinylated anti-human VCAM-1 (PharMingen) (1 µg/mL) for 1 hour. Cells were then exposed to streptavidin alkaline phosphatase (1∶5000 dilution) and the appropriate substrate.

### Serum Biomarker Analyses

The collected blood samples were layered over previously prepared Ficoll gradients.

Five milliliters of the serum were removed and stored in a properly identified freezer (−70°C).

Serum levels of IL-6 and VEGF were assessed by ELISA with the DuoSet kit (R&D Systems Minneapolis, MN, U.S.) according to manufacturer’s instructions and the analysis protocol.

The serum levels of high sensitivity CRP (hsCRP) were measured by the CardioPhase hsCRP Reagent (Siemens Medical Solutions Diagnostics) according to manufacturer’s instructions.

### Statistical Analysis

Continuous data are presented as medians and inter quartile ranges (25^th^–75^th^ percentiles) for skewed distributed variables or as mean **±** SD when normally distributed, and categorical data are presented as absolute numbers and respective percentages. Chi-square tests were used for categorical variables and Student’s *t* test for continuous variables. Correlation analyses between various parameters were provided using Pearson correlation coefficients. Analyses were considered significant at p≤0.05.

## Results

**Figure 1:**
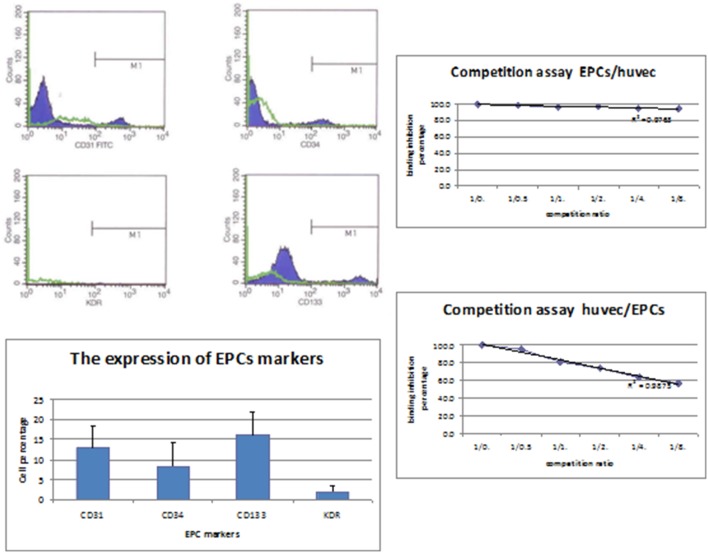
Analysis of late outgrowth EPC and binding characteristics of circulating anti-EPC antibodies. Late outgrowth EPC were obtained from several healthy individuals as described in methods. FACS was used test for surface marker expression (Left panel). To determine if antibodies to EPC are identical to AECA we performed competitive inhibition studies where anti-EPC or AECA were preincubated with EPC or AECA at different ratios and their binding to sold phase bound EPC or HUVEC tested by ELISA as described in methods (Right panel).

Late outgrowth EPC were used for the cyto ELISA as the target cells for the detection of anti-EPC abs as they have been shown to be the most accurate cellular subset to mirror EPC. The markers expressed by this cell population using FACS were: 8.3**±**5.7% to CD34, 12.8**±**5.4% to CD31, 1.9**±**1.4% to KDR and 16.1**±**5.6% to CD133 (Figure 1).

To determine if anti-EPC antibodies are actually AECA were performed competitive inhibition studies. We have found that preincubation of serum harboring anti EPC abs with HUVEC reduced the binding to solid phase bound EPC only by approximately 7% whereas EPC reduced them significantly, suggesting most of these populations are non overlapping.

**Figure 2:**
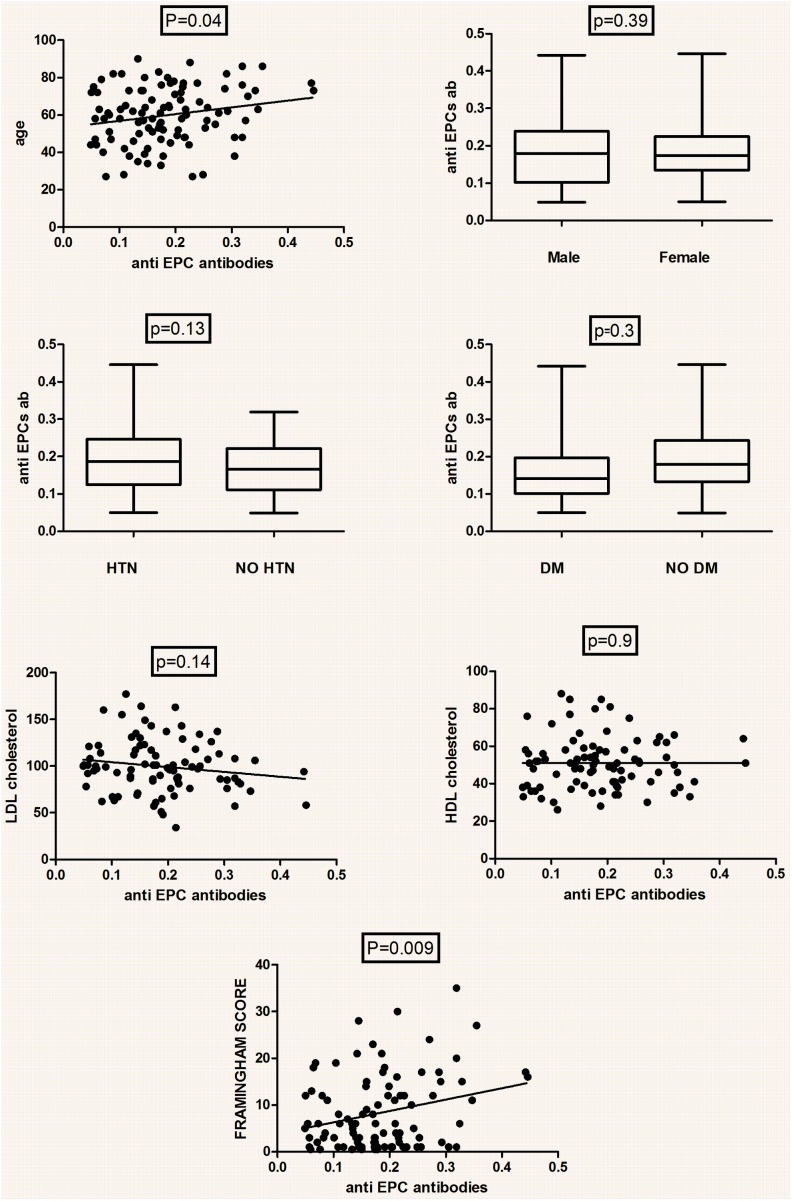
Association between anti-EPC levels and risk factors for atherosclerotic vascular disease. Levels of anti-EPC abs were determined by cyto ELISA as described in methods. The levels were correlated with the presence of individual risk factors (Age, gender, presence of hypertension and diabetes mellitus) and cumulative Framingham score.

Anti-EPC antibody level was associated with the age of the subjects (Figure 2), however no difference in the levels was evident between male versus females nor have been found among diabetics compared with non-diabetic and for hypertensives versus non hypertensives (Figure 2).

Furthermore, levels of anti-EPC abs did not correlate with LDL, TG or HDL levels (Table 2). No association was also detected between antibody levels and IL-6 or VEGF circulating concentrations. However a positive correlation was evident between the levels of anti-EPC abs and circulating hsCRP (Table 2).

**Table 2:** Correlation between anti EPC abs, laboratory results and biomarkers.

	R	P
Hg	−0.15	0.16
WBC	−0.03	0.9
Platelets	−0.06	0.58
Creatinine	0.09	0.37
Total cholesterol	−0.15	0.15
TG	−0.14	0.19
hsCRP	0.29	0.004
VEGF	0.04	0.7
IL-6	0.02	0.8

**Figure 3:**
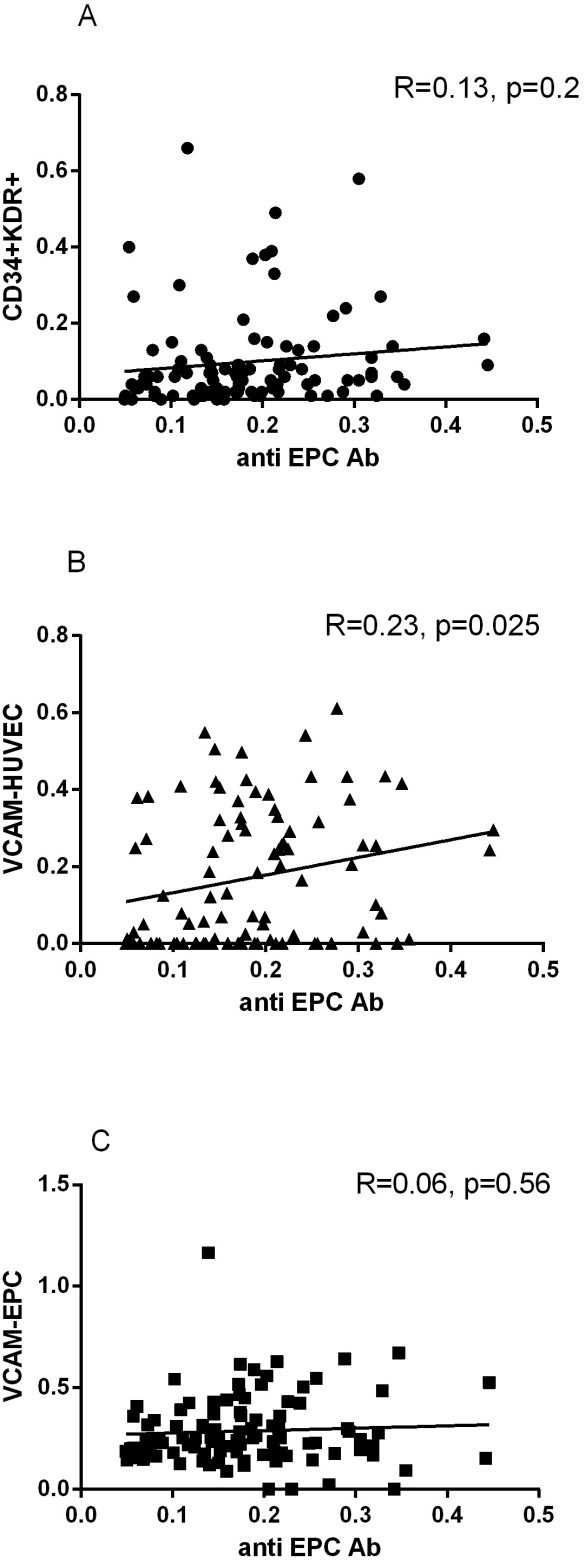
Association of anti-EPC abs with circulating EPC and with VCAM-1 expression on HUVEC and EPC. Serum anti-EPC levels were correlated with circulating CD34+KDR+EPC (A) and with surface expression of the adhesion molecule VCAM-1 on HUVEC (B) and late outgrowth EPC (C).

A significant correlation was found between the levels of anti-EPC abs and the cumulative Framingham score (Figure 2). Interestingly, no correlation was found between levels of anti-EPC abs and circulating EPC defined as CD34+KDR positive cells (Figure 3A).

Aiming to explore whether anti-EPC abs have potential functional properties, we tested the effect of sera from all subject for their ability to induce VCAM-1 expression on solid phase bound HUVEC and EPC (Figure 3B&C). Indeed, we have found a correlation between levels of anti EPC abs and the extent of EC activation as measured by VCAM-1 expression by HUVEC. No such correlation was evident with regard to VCAM-1 expression on EPC. Mean OD values from HUVEC binding anti-EPC IgG fractions exhibited a significant mean 46% increase in VCAM-1 expression as compared with IgG fractions obtained from non-HUVEC binding sera.

## Discussion

This is the first study documenting the presence of autoantibody reactive with EPC in patients with a graduated atherosclerotic risk profile. We have found that circulating autoantibodies that bind EPC can be found by a simple ELISA similar to that developed for AECA.

AECA has been detected in various connective tissue diseses (SLE, antiphospholipid syndrome, systemic sclerosis) and vasculitides ect [11], [14], [15], [17]. The main obstacle to studying the true nature of AECA, similar to other cell-binding autoantibodies, is their heterogeneity in terms of binding epitopes as well as the lack of standardization of their detection method. With regard to atherosclerosis, small studies have not yielded conclusive results as to the prevalence of AECA [13], [18], yet patients with early or masked hypertension do possess higher titers of these circulating autoantibodies [19], [20].

The interesting aspect of AECA is their potential pathogenic role and in particular their ability to induce endothelial activation by triggering surface expression of adhesion molecules such as VCAM-1 on the surface of EC [14], [15]. Indeed, considerable data has been provided to support this function of AECA [14].

The cumulative observations with regard to AECA led us to speculate that similar to endothelial binding antibodies, anti-EPC autoantibodies are also present. The data we demonstrate herein with regard to circulating EPC and their association with risk factors for atherosclerosis are much more robust that those that exist with circulating AECA and show that lower anti-EPC antibody levels are associated with risk factors of vascular disease [5], [6]. Thus, it can be assumed that the detection of such circulating antibodies may be at least equally important to those of circulating EPC and to AECA.

One of the major hurdles in the study of EPC relates to the wide spectrum of binding antigens expressed by these cells. Accordingly, we have previously shown that different methods used to characterize EPC do not correlate with each other, and the method more closely associated with circulating levels of the EPC-promoting-VEGF is the CD34+KDR+ subpopulation [21]. Herein, we have used for the ELISA, EPC that were recently recognized as true endothelial progenitors, namely late outgrowth EPC and also verified by FACS their co-expression of CD34 and CD31.

It is likely that a portion of anti-EPC antibodies cross-react with endothelial cells as EPC develop into EC. Our competitive inhibition studies support this contention, yet do suggest that AECA and anti-EPC abs are not an identical cellular subsets.

The titers of autoantibody reactive with EPC were increased in subjects with the higher Framingham scores (Figure 2) and higher hsCRP levels. Such a finding has not been described for AECA suggesting that a closer association of anti-EPC antibodies with atherosclerosis may exist. This is further supported by the association that was evident between antibody levels and age, a finding that may also be amplified by the well acknowledged increase in total IgG levels with aging. Interestingly, anti-EPC antibodies are not correlated with EPC numbers suggesting differential control mechanisms governing their production although both correlate with risk factor index for atherosclerosis.

An interesting and provocative question with regard to anti-EPC autoantibodies relate to their potential pathogenic role. For AECA, it has been suggested that several properties could mediate their pathogenic potential including, endothelial activation [14], [15], enhanced coagulation [22] and even triggering EC apoptosis [23]. Although, we cannot provide definite answers to this question, we did notice that sera from subjects with higher titers of anti-EPC antibodies were capable of producing a more pronounced effect on VCAM-1 expression on EC *in vitro* (Figure 3B). If this is occurring in vivo, it could be assumed that EPC autoantibodies may have a role in promoting endothelial dysfunction, with subsequent contribution to inflammatory disorders and atheroma formation. Indeed, Del Papa et al [23] have shown for the first time, that IgG fractions obtained from bone marrow plasma of scleroderma patients exhibited endothelial binding activity that was associated with the extent of EPC apoptosis. In contrast with these observations, anti-EPC abs could represent an epiphenomenal autoimmune response to EPC undergoing apoptotic cell death *in vivo*. We have recently provided evidence that such a population of apoptotic progenitors is present *in vivo* in patients with coronary atherosclerosis [24] and heart failure [25], where oxidative stress is heightened.

In conclusion, we provide for the evidence for the presence of autoantibodies reactive with circulating EPC and that the level of these antibodies correlated with the Framingham risk score for atherosclerotic vascular disease. Further studies are required to test the importance of anti-EPC autoantibodies and their potential pathogenic role in promoting vascular dysfunction.
